# Parameter Identification of Robot Manipulators: A Heuristic Particle Swarm Search Approach

**DOI:** 10.1371/journal.pone.0129157

**Published:** 2015-06-03

**Authors:** Danping Yan, Yongzhong Lu, David Levy

**Affiliations:** 1 College of Public Administration, Huazhong University of Science and Technology, Wuhan, Hubei, China; 2 Non-traditional Security Center of Huazhong University of Science and Technology, Wuhan, Hubei, China; 3 School of Software Engineering, Huazhong University of Science and Technology, Wuhan, Hubei, China; 4 Faculty of Engineering and Information Technologies, University of Sydney, Sydney, New South Wales, Australia; Beihang University, CHINA

## Abstract

Parameter identification of robot manipulators is an indispensable pivotal process of achieving accurate dynamic robot models. Since these kinetic models are highly nonlinear, it is not easy to tackle the matter of identifying their parameters. To solve the difficulty effectively, we herewith present an intelligent approach, namely, a heuristic particle swarm optimization (PSO) algorithm, which we call the elitist learning strategy (ELS) and proportional integral derivative (PID) controller hybridized PSO approach (ELPIDSO). A specified PID controller is designed to improve particles’ local and global positions information together with ELS. Parameter identification of robot manipulators is conducted for performance evaluation of our proposed approach. Experimental results clearly indicate the following findings: Compared with standard PSO (SPSO) algorithm, ELPIDSO has improved a lot. It not only enhances the diversity of the swarm, but also features better search effectiveness and efficiency in solving practical optimization problems. Accordingly, ELPIDSO is superior to least squares (LS) method, genetic algorithm (GA), and SPSO algorithm in estimating the parameters of the kinetic models of robot manipulators.

## Introduction

Until now, robot manipulators have been used in a wide variety of industrial engineering projects, ranging from material handling and assembly to cutting, wedding, gluing, and painting. To achieve productivity and flexibility in fully automated production lines, robot manufactures invest much more time and effort in developing advanced model-based control schemes. Dynamic robot models are crucial in these developments because they can be used to linearize nonlinear robot systems in both joint spaces and task spaces. Moreover, the design of advanced model-based robot controllers are usually based on these models and their performances are directly related to the accurate dynamics of the robot systems. Although the dynamic robot models are well known, the dynamic parameters are not easily available since they are not often provided by robot manufactures and are not directly measurable in practice due to the structural complexity and payload of robot manipulators. Therefore, the subject of estimating the model parameters has drawn the aspiring attention from many researchers and practitioners around the world. They have been committed to the study of robot identification for decades of years.

A good number of effectively theoretical and experimental methods to obtain accurate dynamic parameters of robot manipulators have been recently reported in the literatures. These methods may be categorized as on-line identification and off-line identification methods. There are three main off-line identification methods for estimating dynamic parameters of robot manipulators: (1) Physical experiments of dismantled robot manipulators; (2) Computer aided design and manufacturing packages based techniques by using the geometric and material characteristics of robot manipulators; (3) Input/output behavior matching based method: It is deemed as the best choice of these off-line identification methods due to its better identification accuracy and easier measurement [1]. However, the on-line identification methods consist of the following two categories: (1) Traditional cybernetic methods: These methods include a wide variety of solutions such as step response function, pulse response function, frequency response method, correlation analysis method, spectrum analysis method, LS method, and maximum likelihood method, and so on. (2) Modern intelligent optimization techniques: These methods mainly depend on intelligent algorithms and optimization theories to deal with the parameter estimation of robot manipulators. The typical ones of them include neural networks, GA, PSO, differential evolution, set membership method, and other intelligent algorithms and optimization methods.

Among the above methods for the parameter estimation of robot manipulators, traditional cybernetic methods are usually employed to obtain accurate dynamic parameters [2–15]. In [2], Billings and Fakhouri employed cross-correlation techniques to decouple the identification of the linear dynamics from the characterization of the nonlinear element in the nonlinear systems when the input is a white Gaussian signal [2]. Gautier and Khalil presented a direct method for determining the minimum set of inertial parameters of serial robots. The method contributes to the reduction of the computational cost of the dynamic models and simplifies the identification of the inertial parameters by using least squares method [3]. To overcome the difficulties of noise on position and torque measurements, friction modeling error and bad excitation, Vandanjon, Gautier, and Desbats proposed a new identification method based on experiments which are designed by means of physical interpretation and spectrum analysis of the robot dynamic model in order to reduce sensitivity to noise. The successful application of this new method to a 3 degrees of freedom robot proves the efficiency of the algorithms [4]. Olsen and Petersen developed a new maximum likelihood method for estimating inertial parameters. The experiments in their study were carried out on the first two links of a seven-axis Mitsubishi PA-10 robot in [5]. It is worth noting that although the least squares method is regarded as the most widely used one, there exist some problems of estimating the accurate dynamic parameters by using it. It can often generate the solutions with temporary minima or non-optimal local minima and it is also sensitive to measurement noise. One can use the so-called exciting trajectory that can guarantee the excitation of all the dynamic parameters to be identified or use the data filtering to overcome the measurement noise sensitivity [6]. Furthermore, one promising solution for the problem of the so-called physical feasibility of the identified parameters is to use constrained optimization tools to adjust the least squares results [7–10]. Gautier, Janot, and Vandanjon presented a closed-loop output error method where the usual joint position output is replaced by the joint force/torque. Its merit is to avoid the calculation of the velocity and acceleration by bandpass filtering of the measured position. The method is experimentally validated on an industrial Stäubli RX-90 robot and a two degree-of-freedom direct drive rigid robot [11]. In addition, Díaz-Rodríguez, Mata, Valera, and Valera put forward a strategy for dynamic parameter identification of parallel robots in terms of relevant parameters. The dynamic model developed by means of the Gibbs-Appell equations is simplified based on the considered geometry of each link and symmetry of the legs. The identification is done by Weighted Least Squares. The strategy has been experimentally tested on two actual 3-DOF parallel robots [12]. Calanca, Capisani, Ferrara, and Magnani proposed a practical multi-input multi-output (MIMO) closed loop parameters identification procedure for robot manipulators. It is based on the weighted least squares method. The presented procedure has been successfully experimentally tested on a COMAU SMART3-S2 industrial manipulator used in a planar configuration [13]. In [14], Thanh, Kotlarski, Heimann, and Ortmaier addressed both modelling and dynamics identification of kinematically redundant parallel robots, based on the Lagrangian equations of the first kind and the coordinate partitioning method. The dynamic parameter estimation of an exemplarily considered kinematically redundant 3-(P)RRR parallel robot using a least-squares approach and a nonlinear optimization technique is discussed. More recently, Janot, Vandanjon, and Gautier proposed an instrumental variable method for robot identification. The instrument set is the inverse dynamic model built from simulated data calculated from simulation of the direct dynamic model. The simulation is based on previous estimates and assumes the same reference trajectories and the same control structure for both actual and simulated robots. The gains of the simulated controller are updated according to instrumental variable estimates to obtain a valid instrument set at each step of the algorithm. The proposed approach validates the inverse and direct dynamic models simultaneously, is not sensitive to initial conditions, and converges rapidly. Experimental results obtained on a six degrees-of-freedom industrial robot show the effectiveness of this approach [15].

Over the last two decades, with the advance of intelligent algorithms and optimization theories, modern intelligent optimization techniques have been playing an increasingly important role in the field of parameter identification of robot manipulators [16–20]. Khemaissia and Morris addressed the novel issues related to system identification of robot manipulators based on the nonlinear functional properties of artificial neural network models. An estimation procedure for the link parameters is described in which identification is carried out using the parallel recursive prediction error technique. The algorithm enables the weights in each neuron of the network to be updated in an efficient parallel manner and has better convergence than the classical back propagation algorithm. The whole of the algorithm can be distributed over a network of parallel processors to achieve impressive speed-up. An example is given for the first three links of the Stanford arm to demonstrate the effectiveness of this algorithm [16]. Besides, Anh used a novel inverse dynamic MIMO NARX model for modeling and identifying simultaneously both of joints of the prototype 2-axes PAM robot arm. The contact force variations and highly nonlinear coupling features of both links of the 2-axes PAM system are modeled thoroughly through an inverse neural MIMO NARX model-based identification process using experiment input-output training data. For the first time, the dynamic inverse neural MIMO NARX model of the 2-axes PAM robot arm has been investigated. The results show that the neural inverse dynamic MIMO NARX model trained by back propagation learning algorithm yields outstanding performance and perfect accuracy [17]. Bingül and Karahan deal with the dynamic modeling and identification of Stäubli RX-60 robot by least squares method and PSO technique. These experimental results show that the estimated inertial parameters predict robot dynamics well [18]. Supriyono and Tokhi presented current biologically-inspired optimization techniques and their application to modeling of a single-link flexible manipulator [19]. Köker proposed a hybrid approach which combines the characteristics of neural networks and evolutionary techniques to obtain more precise solutions. The neural networks and genetic algorithms are used together to solve the inverse kinematic problem of a six-joint Stanford robotic manipulator to minimize the error at the end effector [20].

Being different from the traditional cybernetic methods, the modern intelligent algorithms are not sensitive to the complex characteristics of the kinetic models of robot manipulators and are able to promptly search the solutions to problems solving in the nonlinearly multi-dimensional spaces. As a result, we herewith attempt to pursue an effectively intelligent algorithm to solve the inverse kinematic problem of robot manipulators. After an in-depth investigation into the nature of the PSO algorithm, we put forward a novel PSO algorithm which we call the elitist learning strategy (ELS) and proportional integral derivative (PID) controller hybridized PSO approach (ELPIDSO). A specified PID controller is designed to improve particles’ local and global positions information together with ELS. In order to verify the effectiveness of ELPIDSO, we apply it to identify the parameters of the kinetic model of a robot manipulator with two links. Experimental results prove that it is superior to PSO, LS and GA in estimating the parameters of the kinetic models of robot manipulators and that it is a more efficient approach to system identification in real practice.

The rest of the paper is organized as follows. Section 2 describes the kinetic modelling of a two-link robot manipulator and the problem formulation of its parameter identification, and depicts the derivation of ELPIDSO and its ELS, mutation mechanism and the whole procedure. Section 3 presents the experimental study of applying ELPIDSO to the parameter identification of the two-link robot manipulator. Section 4 gives the conclusions and future work.

## Analysis and Methods

In this part, we describe the kinetic modeling and identification criterion of two-link robot manipulator, discuss the stability of ELPIDSO, design a PID controller, depict the ELS and mutation mechanism, and give a full description of the procedure of ELPIDSO in turn.

### Kinetic modeling and identification criterion of two-link robot manipulator

The Lagrangian method may be used to derive the following kinetic equation of kinematic chains of rigid bodies [21, 22]

$$\begin{array}{c} H\left(q,\lambda \right)\ddot{q}+C\left(q,\dot{q},\lambda \right)\dot{q}+G\left(q,\lambda \right)=\tau , \end{array}$$ (1)

which expresses, for an n-degree-of-freedom robot, the n-vector of actuator torques *τ* as a function of the n-vectors of the joint positions q, velocities $\dot{q}$, and accelerations $\overset{..}{q}$ as well as the barycentric parameters *λ* of the model. In Eq (1), **H**(**q**,*λ*) is the *n* × *n* inertia matrix, $\text{C}(\text{q},\dot{\text{q}},\lambda )$ is the *n* × *n* Coriolis and centrifugal matrix, and **G**(**q**,*λ*) represents gravitational torques. **H**(**q**,*λ*), $\text{C}(\text{q},\dot{\text{q}},\lambda )$, and **G**(**q**,*λ*) are nonlinear functions of the model parameters *λ* which include the mass, center-of-gravity location, and moments and products of inertia of each link. The actuator torques **τ** is also expressed by the following equation

$$\begin{array}{c} \tau =Y(q,\dot{q},\ddot{q})\lambda , \end{array}$$ (2)

which is linear in the unknown parameters. In Eq (2)), *λ* is the barycentric parameter vector, and $Y(q,\dot{q},\overset{..}{q})$ is the observation or identification matrix which depends only on the motion data. This property simplifies the parameter estimation considerably. The barycentric parameters of a link are combinations of the inertial parameters of the link and its descendants in the kinematic chain. In general, a robot manipulator with multi-link is better than that with single link because it has multi-joints and is more stable in practice.

**Fig 1:**
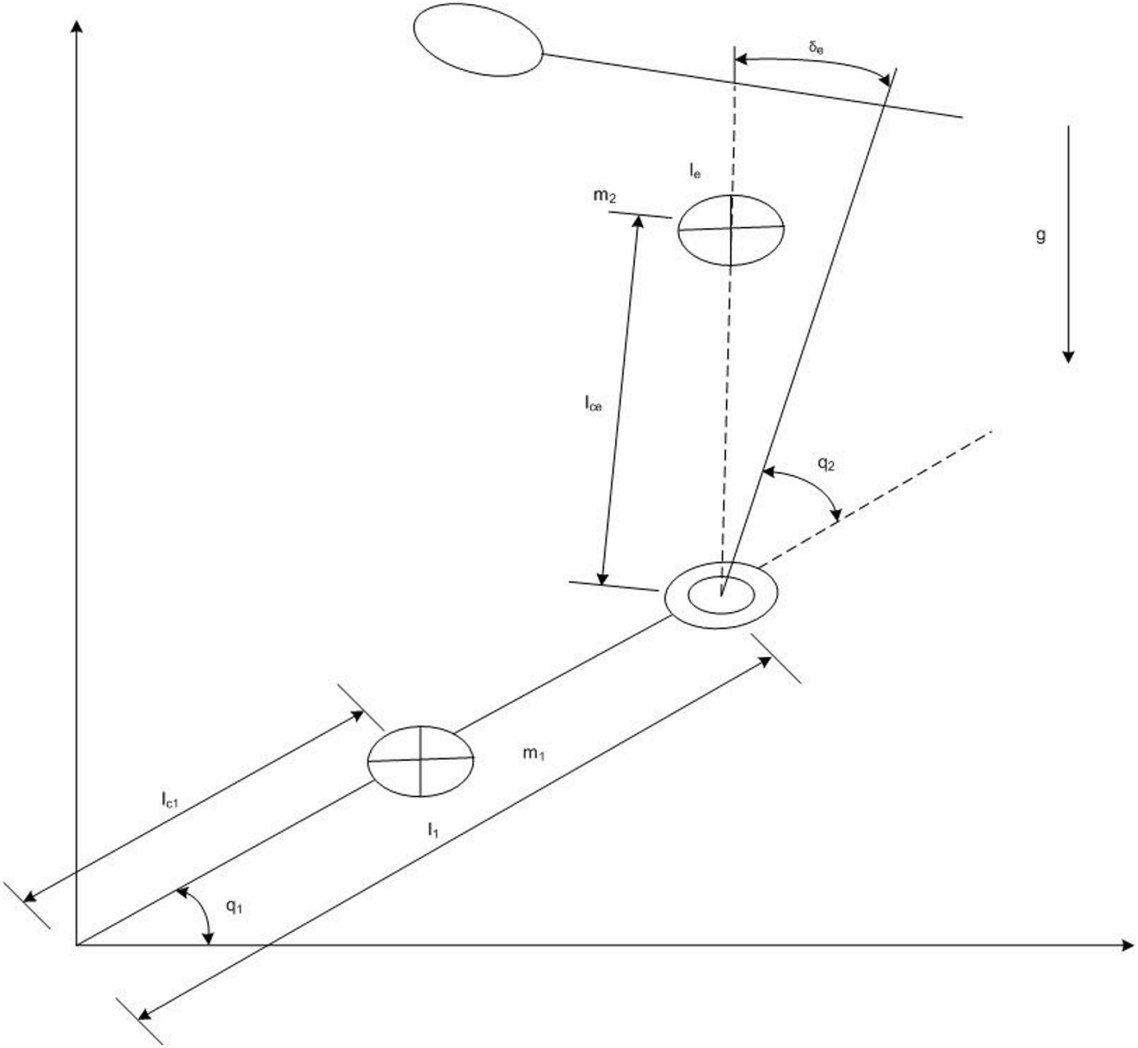
Schematic planar diagram of a two-link robot manipulator with unknown payload.

In particular, for a 2-degree-of-freedom robot manipulator as shown in Fig 1 [23, 24], **H**(**q**,*λ*), $\text{C}(\text{q},\dot{\text{q}},\lambda )$, **G**(**q**,*λ*), $\text{Y}(\text{q},\dot{\text{q}},\overset{..}{\text{q}})$ and *λ* are deduced respectively

$$\begin{array}{c} H(q,\lambda )=[H_{11},H_{12};H_{21},H_{22}], \end{array}$$

$$\begin{array}{c} C(q,\dot{q},\lambda )=\left[C_{11},C_{12};C_{21},C_{22}\right], \end{array}$$

$$\begin{array}{c} G(q,\lambda )=[G_{11};G_{21}], \end{array}$$

$$\begin{array}{c} Y(q,\dot{q},\ddot{q})=\left[Y_{11},Y_{12},Y_{13},Y_{14};Y_{21},Y_{22},Y_{23},Y_{24}\right], \end{array}$$

$$\begin{array}{c} \lambda =[\alpha ;\beta ;\varepsilon ;\eta ], \end{array}$$

where *H*_11_, *H*_12_, *H*_21_, *H*_22_, *C*_11_, *C*_12_, *C*_21_, *C*_22_, *G*_11_, *G*_21_, *Y*_11_, *Y*_12_, *Y*_13_, *Y*_14_, *Y*_21_, *Y*_22_, *Y*_23_, *Y*_24_, *e*_1_, *e*_2_, *g*, *α*,*β*,*ɛ* *and* *η* are defined as follows,

*H*_11_ = *α*+2*ɛcos*(*q*_2_)+2*ηsin*(*q*_2_),
*H*_12_ = *β*+*ɛcos*(*q*_2_)+*ηsin*(*q*_2_),
*H*_21_ = *β*+*ɛcos*(*q*_2_)+*ηsin*(*q*_2_),
*H*_22_ = *β*,
$C_{11}=(-2ɛsin(q_{2})+2\eta cos(q_{2}))\dot{q_{2}}$,
$C_{12}=(-ɛsin(q_{2})+\eta cos(q_{2}))\dot{q_{2}}$,
$C_{21}=(ɛsin(q_{2})-\eta cos(q_{2}))\dot{q_{1}}$,
*C*_22_ = 0,
*G*_11_ = *ɛe*_2_ *cos*(*q*_1_+*q*_2_)+*ηe*_2_ *sin*(*q*_1_+*q*_2_)+(*α*−*β*+*e*_1_)*e*_2_ *cos*(*q*_1_)),
*G*_21_ = *ɛe*_2_ *cos*(*q*_1_+*q*_2_)+*ηe*_2_ *sin*(*q*_1_+*q*_2_),
$Y_{11}=\overset{..}{q_{1}}+e_{2}cos(q_{1})$,
$Y_{12}=\overset{..}{q_{2}}-e_{2}cos(q_{1})$,
$Y_{13}=2cos(q_{2})\overset{..}{q_{1}}+cos(q_{2})\overset{..}{q_{2}}-2sin(q_{2})\dot{q_{2}}\dot{q_{1}}-sin(q_{2})\dot{q_{2}}\dot{q_{2}}+e_{2}cos(q_{1}+q_{2})$,
$Y_{14}=2sin(q_{2})\overset{..}{q_{1}}+sin(q_{2})\overset{..}{q_{2}}+2cos(q_{2})\dot{q_{2}}\dot{q_{1}}+cos(q_{2})\dot{q_{2}}\dot{q_{2}}+e_{2}sin(q_{1}+q_{2})$,
*Y*_21_ = 0,
$Y_{22}=\overset{..}{q_{1}}+\overset{..}{q_{2}}$,
$Y_{23}=cos(q_{2})\overset{..}{q_{1}}+sin(q_{2})\dot{q_{1}}\dot{q_{1}}+e_{2}cos(q_{+}q_{2})$,
$Y_{24}=sin(q_{2})\overset{..}{q_{1}}-cos(q_{2})\dot{q_{1}}\dot{q_{1}}+e_{2}sin(q_{+}q_{2})$,

$e_{1}=m_{1}l_{1}l_{c1}-I_{1}-m_{1}l_{1}^{2}$, *e*_2_ = *g*/*l*_1_, *g* is the gravitational acceleration, $\alpha =I_{1}+m_{1}l_{c1}^{2}+I_{e}+m_{e}l_{ce}^{2}+m_{e}l_{1}^{2}$, $\beta =I_{e}+m_{e}l_{ce}^{2}$, *ɛ* = *m*_*e*_ *l*_1_ *l*_*ce*_ *cos*(*δ*_*e*_) and *η* = *m*_*e*_ *l*_1_ *l*_*ce*_ *sin*(*δ*_*e*_).

Since $\text{Y}(\text{q},\dot{\text{q}},\overset{..}{\text{q}})$ is highly nonlinear and the parameters in *λ* are linearly independent, some effective approaches like LS, GA, SPSO and so on are often used for identifying the barycentric parameter vector *λ*. During the course of the parameter estimation, the identification criterion function is generally defined below

$$\begin{array}{c} E=\sum_{i=1}^{N}\frac{1}{2}{\left(\tau_{i}-\hat{\tau_{i}}\right)}^{T}\left(\tau_{i}-\hat{\tau_{i}}\right), \end{array}$$ (3)

where *N* is the number of testing samples, *τ*_*i*_ is the measured torque value of the *ith* testing sample, and $\hat{\tau_{i}}$ is the estimated prediction torque value of the *ith* testing sample.

### Analyzing the stability of ELPIDSO and designing a PID controller

PSO is a stochastic population-based algorithm which is modeled on the behaviors of insects swarming, animals herding, birds flocking, and fish schooling where these swarms search for food in a collaborative manner, and it was originally introduced by Kennedy and Eberhart in 1995 [25, 26]. It is usually used for the optimization of continuous nonlinear systems. Since PSO uses a simple swarm emulating mechanism to guide the particles to search for globally optimal solutions and performs easily, it has succeed in solving many real-world optimization problems [27–42].

Similar to other evolutionary computation algorithms, the SPSO algorithm also shares a population-based iterative evolution technique. Hence, it can computationally be inefficient as measured by the number of function evaluations (FEs) required. Moreover, it may easily get trapped in the local optimum when solving complex multimodal problems. In order to improve the performance of the SPSO algorithm and achieve the specific goals of accelerating convergence speed and avoiding local optima, a number of variants of the SPSO algorithm have been proposed so far in spite of being difficult to simultaneously demonstrate these expectations. We herein bring forward a novel PSO approach called ELPIDSO.

SPSO is a kind of typically stochastic standard algorithm to search for the best solution by simulating the movement of the flocking of birds or fish. It works by initializing a flock of birds or fish randomly over the searching space, where each bird or fish is called a “particle”. These particles fly with certain velocities and find the global best position after some generations. At each generation, they are dependent on their own momentum and the influence of their own local and global best positions *x*_*lbest*_ and *x*_*gbest*_ to adjust their own next velocity *v* and position *x* to move in turn. SPSO is clearly depicted as follows

$$\begin{array}{c} v\left(t+1\right)=\omega_{pso}\cdot v\left(t\right)+c_{1}\cdot rand_{1}\cdot \left(x_{lbest}-x\left(t\right)\right)+c_{2}\cdot rand_{2}\cdot \left(x_{gbest}-x\left(t\right)\right), \end{array}$$ (4)

$$\begin{array}{c} x(t+1)=v(t+1)+x(t), \end{array}$$ (5)

where *ω*_*pso*_,*c*_1_ and *c*_2_ denote the inertia weight coefficient, cognitive coefficient and social coefficient, respectively, and *rand*_1_,*rand*_2_ are both random values between 0 and 1. Besides, *v* is clamped to a given range [-*v*_*max*_,+*v*_*max*_].

Adjusting Eqs (4) and (5) to Eqs (6) and (7)

$$\begin{array}{ccc} v(t+1)-v(t) & = & \left(\omega_{pso}-1\right)\cdot v\left(t\right)-\left(c_{1}\cdot rand_{1}+c_{2}\cdot rand_{2}\right)\cdot x\left(t\right)\\ & & +\;(c_{1}\cdot rand_{1}\cdot x_{lbest}+c_{2}\cdot rand_{2}\cdot x_{gbest}), \end{array}$$ (6)

$$\begin{array}{ccc} x(t+1)-x(t) & = & v(t+1)\\ & = & \omega_{pso}\cdot v\left(t\right)-\left(c_{1}\cdot rand_{1}+c_{2}\cdot rand_{2}\right)\cdot x\left(t\right)\\ & & +\;(c_{1}\cdot rand_{1}\cdot x_{lbest}+c_{2}\cdot rand_{2}\cdot x_{gbest}). \end{array}$$ (7)

Supposing *ϕ*_1_ = *c*_1_ ⋅ *rand*_1_, *ϕ*_2_ = *c*_2_ ⋅ *rand*_2_, *ϕ* = *c*_1_ ⋅ *rand*_1_+*c*_2_ ⋅ *rand*_2_ and $\theta =\frac{\phi_{2}}{\phi }$, Eqs (6) and (7) can be transformed into the following differential evolutionary SPSO Eqs (8) and (9) since $v(t+1)-v(t)\approx \frac{dv}{dt}$ and $x(t+1)-x(t)\approx \frac{dx}{dt}$

$$\begin{array}{c} \frac{dv}{dt}\approx \left(\omega_{pso}-1\right)\cdot v\left(t\right)-\phi \cdot x\left(t\right)+\left(\phi_{1}\cdot x_{lbest}+\phi_{2}\cdot x_{gbest}\right), \end{array}$$ (8)

$$\begin{array}{c} \frac{dx}{dt}\approx \omega_{pso}\cdot v\left(t\right)-\phi \cdot x\left(t\right)+\left(\phi_{1}\cdot x_{lbest}+\phi_{2}\cdot x_{gbest}\right). \end{array}$$ (9)

Provided the initial outsets *V*(0) ≈ 0 *and* *X*(0) ≈ 0, the following formulae are obtained after the Laplace transformation of differential evolutionary SPSO equations

$$\begin{array}{c} V\left(s\right)\approx \frac{s}{s+1}\cdot X\left(s\right), \end{array}$$ (10)

$$\begin{array}{ccc} X(s) & \approx & \frac{\phi_{1}\cdot \left(s+1\right)}{s^{2}+s\cdot \left(1-\omega_{pso}+\phi \right)+\phi }\cdot X_{lbest}\left(s\right)+\frac{\phi_{2}\cdot \left(s+1\right)}{s^{2}+s\cdot \left(1-\omega_{pso}+\phi \right)+\phi }\cdot X_{gbest}\left(s\right)\\ & = & \frac{\phi_{1}\cdot \left(s+1\right)}{s\cdot (s+1-\omega_{pso})}\cdot \left(X_{lbest}\left(s\right)-X\left(s\right)\right)+\frac{\phi_{2}\cdot \left(s+1\right)}{s\cdot (s+1-\omega_{pso})}\cdot \left(X_{gbest}\left(s\right)-X\left(s\right)\right)\\ & = & \frac{\phi \cdot (s+1)}{s\cdot (s+1-\omega_{pso})}\cdot \left(\left(\left(1-\theta \right)\cdot X_{lbest}\left(s\right)+\theta \cdot X_{gbest}\left(s\right)\right)-X\left(s\right)\right). \end{array}$$ (11)

Supposing $G(s)=\frac{\phi \cdot (s+1)}{s\cdot (s+1-\omega_{pso})}$, the closed-loop transfer function for the input *X*(*s*) and the combination output of *X*_*lbest*_(*s*) and *X*_*gbest*_(*s*) is the following formula (12)

$$\begin{array}{c} \frac{X(s)}{\left(\left(1-\theta \right)\cdot X_{lbest}\left(s\right)+\theta \cdot X_{gbest}\left(s\right)\right)}=\frac{G(s)}{1+G(s)}. \end{array}$$ (12)

Provided *X*_*lbest*_(*s*) = *X*_*gbest*_(*s*), Eq (12) is changed into Eq (13)

$$\begin{array}{c} \frac{X(s)}{X_{gbest}\left(s\right)}=\frac{G(s)}{1+G(s)}. \end{array}$$ (13)

**Fig 2:**
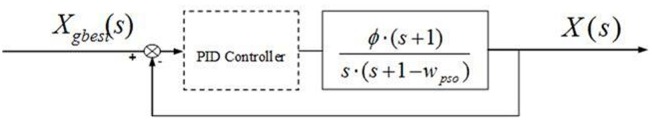
The relationship between the position and the global best position.

Thus, the evolutionary relationship between the position *X*(*s*) and its global best position *X*_*gbest*_(*s*) in a closed-loop scheme is displayed in Fig 2. It clearly illustrates their evolutionary relationship which denotes a second order transfer function. In order to advance the evolutionary relationship, we add one PID controller between *X*_*gbest*_(*s*) and *X*(*s*) as in Fig 2, where the PID controller is expressed by Eq (14) [43]

$$\begin{array}{c} G_{PID}\left(s\right)=k_{p}+\frac{k_{i}}{s}+k_{d}\cdot s \end{array}$$ (14)

and appears in the dashed framework.

Accordingly, we obtain the following formula (15)

$$\begin{array}{c} \frac{X(s)}{X_{gbest}\left(s\right)}=\frac{G_{PID}\left(s\right)\cdot G\left(s\right)}{1+G_{PID}\left(s\right)\cdot G\left(s\right)}. \end{array}$$ (15)

The corresponding eigenvalue function is expressed below by Eq (16)

$$\begin{array}{c} 1+G_{PID}\left(s\right)\cdot G\left(s\right)=0, \end{array}$$ (16)

namely,

$$\begin{array}{c} \left(1+k_{d}\cdot \phi \right)\cdot s^{3}+\left(1+k_{p}\cdot \phi +k_{d}\cdot \phi \right)\cdot s^{2}+\left(-\omega_{pso}+\phi \cdot k_{p}+\phi \cdot k_{i}\right)\cdot s+k_{i}\cdot \phi =0. \end{array}$$

According to Routh-Hurwitz’s stability criterion, the inequalities are obtained below

$$\begin{array}{c} -\omega_{pso}+k_{p}\cdot \phi +k_{d}\cdot \phi >0, \end{array}$$ (17)

$$\begin{array}{c} \left(k_{p}^{2}+k_{i}\cdot k_{p}+k_{d}\cdot k_{p}\right)\cdot \phi^{2}+\left(k_{p}-\omega_{pso}\cdot \left(k_{p}+k_{d}\right)\right)\cdot \phi -\omega_{pso}>0. \end{array}$$ (18)

On the other hand, the updated *X*(*s*) is as follows

$$\begin{array}{ccc} X(s) & = & \frac{\phi_{1}\cdot \left(s+1\right)\cdot G_{PID}\left(s\right)}{s\cdot (s+1-\omega_{pso})}\cdot \left(X_{lbest}\left(s\right)-X\left(s\right)\right)+\frac{\phi_{2}\cdot \left(s+1\right)\cdot G_{PID}\left(s\right)}{s\cdot (s+1-\omega_{pso})}\cdot \left(X_{gbest}\left(s\right)-X\left(s\right)\right)\\ & = & \frac{\phi \cdot \left(s+1\right)\cdot G_{PID}\left(s\right)}{s\cdot (s+1-\omega_{pso})}\cdot \left(\left(\left(1-\theta \right)\cdot X_{lbest}\left(s\right)+\theta \cdot X_{gbest}\left(s\right)\right)-X\left(s\right)\right). \end{array}$$ (19)

After being combined with Eqs (19) and (10) is presented below

$$\begin{array}{c} V\left(s\right)=\frac{\phi \cdot G_{PID}\left(s\right)}{s\cdot (s+1-\omega_{pso})}\cdot \left(\left(\left(1-\theta \right)\cdot X_{lbest}\left(s\right)+\theta \cdot X_{gbest}\left(s\right)\right)-X\left(s\right)\right). \end{array}$$ (20)

Thus, Eq (20) is turned into the following time-varying function formula (21) after the inverse Laplace Transformation

$$\begin{array}{ccc} v(t+1) & = & \omega_{pso}\cdot v\left(t\right)\\ & & +\;\phi \cdot (\left(1-\theta \right)\cdot \left(k_{p}\cdot \left(x_{lbest}-x\left(t\right)\right)+k_{i}\cdot \int_{0}^{t}\left(x_{lbest}-x\left(t\right)\right)\cdot dt+k_{d}\cdot \frac{d(x_{lbest}-x\left(t\right))}{dt}\right)\\ & & +\;\theta \cdot (k_{p}\cdot \left(x_{gbest}-x\left(t\right)\right)+k_{i}\cdot \int_{0}^{t}\left(x_{gbest}-x\left(t\right)\right)\cdot dt+k_{d}\cdot \frac{d(x_{gbest}-x\left(t\right))}{dt})). \end{array}$$ (21)

Consequently, our proposed ELPIDSO is comprised of Eqs (21) and (5).

Being different from Eq (4) in SPSO, Eq (21) not only includes the proportional terms of (*x*_*lbest*_−*x*(*t*)) and (*x*_*gbest*_−*x*(*t*)), but also encompasses their integral terms and derivative terms. These terms enable ELPIDSO to achieve a proper response, eliminate the steady-state errors, and improve particles’ evolutionary dynamics simultaneously so that ELPIDSO enhances the diversity of the swarm and converges fast to the global best position.

Based on the above in-Eqs (17) and (18) and our professional experiences, we design the following three coefficients of the PID controller

$$\begin{array}{ccc} k_{p} & = & e^{\left(\omega_{pso}-1\right)\cdot \frac{t}{MaxT}}, \end{array}$$ (22)

$$\begin{array}{ccc} k_{i} & = & \frac{e^{\left(\omega_{pso}-1\right)\cdot \frac{t}{MaxT}}}{1+e^{\left(\omega_{pso}-1\right)\cdot \frac{t}{MaxT}}}, \end{array}$$ (23)

$$\begin{array}{ccc} k_{d} & = & {\left[e^{\left(\omega_{pso}-1\right)\cdot \frac{t}{MaxT}}\right]}^{2}, \end{array}$$ (24)

where *t* is the present generation, and *MaxT* is the maximum generation.

Concerning the inertia weight coefficient, we adopt the following formula (25) [44]

$$\begin{array}{c} \omega_{pso}=\frac{1}{1+1.5\cdot e^{\left(-2.6\cdot f\right)}}, \end{array}$$ (25)

where *f* is supposed to decrease linearly from 1 to 0. In addition, the cognitive coefficient is supposed to decrease linearly from 2 to 0 while the social coefficient is supposed to increase linearly from 0 to 2.

### Elitist learning strategy of ELPIDSO

In order to promote the evolutionary process of ELPIDSO, we adopt an ELS to help particles perform comprehensive learning from their own local and neighboring best positions, and other local best positions. It is evident that particles are easily trapped into local optima after some certain iterations. Therefore, we depend on a learning probability *P*_*c*_*i*__ which can take different values for different particles to enable each dimension of a specific local best particle to learn from the same dimension of an elitist particle from either itself or the hybridization of the local best particles of the population. If a generated random number is larger than *P*_*c*_*i*__, the corresponding dimension will learn from itself; Otherwise, it will learn from the another elitist particle from the better one of two randomly chosen hybridizers of the local best particles. The tournament selection procedure of the another elitist particle is as follows. We first randomly choose two hybridizers from the local best particles. These hybridizers can generate new positions in the search space using the information derived from different local best particles’ historical positions. Then we compare their fitness values and select the better one as the another elitist particle. We repeat the same operation on every dimension of the specific local best particle. As a result, the specific local best position has improved a lot by the ELS. And Eq (21) of ELPIDSO gets more robust and adaptive since the diversity of the population is obviously enhanced. The aforementioned learning probability *P*_*c*_*i*__ for the ith particle is empirically developed below [45]

$$\begin{array}{c} P_{c_{i}}=0.0+\left(0.5-0.0\right)\frac{\left(e^{\frac{5\cdot (i-1)}{PN-1}}-1\right)}{\left(e^{5}-1\right)}, \end{array}$$ (26)

where *PN* is the number of particles.

### Mutation of ELPIDSO

It has been observed that the normal PSO is easily stagnated in local optimum because of the lack of diversity of the population. Thus, particles remain in a local optimum for unpredictable generations. In order to increase search diversity and avoid getting trapped in local optimum, many leaping-out mechanisms are proposed [44–48]. However, the performance can be affected by many factors and is hard to predict after introducing the leaping-out algorithms.

In ELPIDSO, we first randomly select the local best particle (*x*_*lbest*_(*k*)) out of the population. If it is not the global best particle (*x*_*gbest*_), we randomly choose one dimension (*j*) from the selected particle, whose position is $x_{lbest}^{j}(k)$ and velocity vector is $v_{lbest}^{j}(k)$. Thereafter, we use $x_{lbest}^{j}(k)$ to replace the same dimension (*j*) of the global best particle as a temporary global best particle (*x*_*gbest*′_). Otherwise, we use the following equation

$$\begin{array}{c} x_{lbest}^{j}\left(k\right)+\left(x_{max}^{j}-x_{min}^{j}\right)\cdot Gaussian\left(\mu ,\sigma^{2}\right) \end{array}$$ (27)

to do it, where the search range $\left[x_{min}^{j},x_{max}^{j}\right]$ is the same as the lower and upper bounds of the problem, and the Gaussian(*μ*,*σ* ^2^) is a random number of a Gaussian distribution with a zero mean *μ* and a standard deviation *σ*. Similar to some time-varying neural network training schemes, it is suggested that *σ* be linearly decreased with the generation number, which is given by

$$\begin{array}{c} \sigma =1-t/MaxT, \end{array}$$ (28)

where *t* is the present generation and *MaxT* is the maximum generation. Next, we compare the fitness(*x*_*gbest*_) with the fitness(*x*_*gbest*′_). If the fitness(*x*_*gbest*_) > the fitness(*x*_*gbest*′_), the position of the global best particle in the dimension *j* is moved to $x_{gbest^{\prime }}^{j}$ and its updated fitness(*x*_*gbest*_) value is equal to the fitness(*x*_*gbest*′_).

### Procedure of ELPIDSO

Consequently, based on the aforementioned contexts, our proposed ELPIDSO can be depicted below in detail.

Step 1: Initialize parameters including the number *PN* of particles, dimensional size *D* of each particle, maximum generation number *MaxT*, initial position *x* and velocity *v* of each particle, inertia weight coefficient *w*_*pso*_, cognitive coefficient *c*_1_, social coefficient *c*_2_, and learning probability *P*_*c*_. Calculate the initial fitness of each particle, and set the initial local best position *x*_*lbest*_ and global best position *x*_*gbest*_.

Step 2: If the specific local optimal value *x*_*lbest*_(*k*) does not evolve for some certain iterations, improve the specific local best position by the above-mentioned ELS. Thereafter, according to Eqs (22)–(24), calculate the three parameters *k*_*p*_,*k*_*i*_ *and* *k*_*d*_ of the PID controller. Then in terms of Eqs (21) and (5), calculate the next velocity *v*(*k*) and position *x*(*k*) of each particle. Next, calculate the fitness of each particle, set the local best position *x*_*lbest*_ and the global best position *x*_*gbest*_. Next, update the global best position *x*_*gbest*_ with the temporary global best mutation position *x*_*gbest*′_ if the fitness(*x*_*gbest*_) > the fitness(*x*_*gbest*′_).

Step 3: Observe if the global best fitness(*x*_*gbest*_) meets the given threshold or not, or observe if the maximum generation number *MaxT* reaches or not. If not, go back to Step 2.

Step 4: Otherwise, the operation can be terminated. Finally, output the global best position *x*_*gbest*_, and its corresponding global best fitness as well as convergent generation number.

The pseudo-code for ELPIDSO is presented below in Algorithm 1.

## Experimental Study and Results

In this part, we conduct a detailed experimental study to identify the parameters of a two-link robot manipulator. The experiment includes the design of the identification procedure, description of the experimental setup, parameter estimation and experimental results as well as model validation and discussion.

### Design of identification procedure

**Fig 3:**
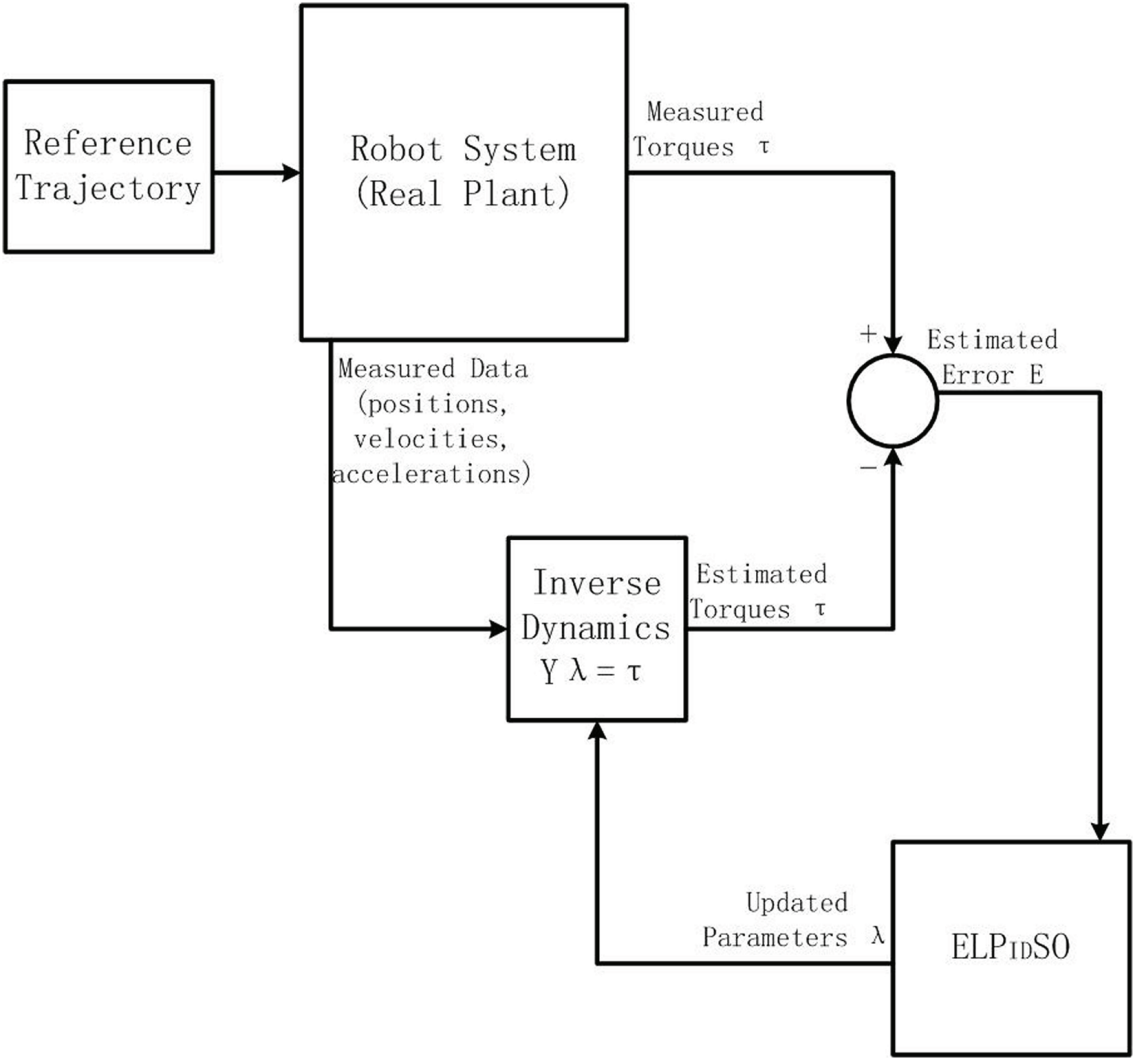
Identification procedure of a two-link robot manipulator with unknown payload.

Fig 3 gives the schematic representation of our experimental robot identification procedure. Note that prior to the identification, the kinematic and geometric information of the robot manipulator and model accuracy specifications are necessarily available because these information determines choices to be made in the procedure. Model accuracy specifications determine the model type to be used and the level of the dynamics to be included in the model. The last step of the identification procedure is the model validation, where the users verify if the model satisfies the accuracy specifications or not. In our procedure, we consider one model validation measure, namely, the actuator torque estimation accuracy. Being different from other experimental robot identification procedures, we introduce ELPIDSO into our experimental identification procedure and take full advantage of it to estimate the parameters of the two-link robot manipulator with unknown payload. Moreover, the well-informed methods used in the identification procedure like LS, GA and PSO are also considered together so as to clarify the effectiveness and efficiency of ELPIDSO.

### Description of experimental setup

The schematic planar structure of the two-link robot manipulator with unknown payload is shown in Fig 1. There exist four unknown physical parameters to be identified in the combination part of the second joint and payload which include the mass *m*_*e*_, moment of inertia *I*_*e*_, distance *l*_*ce*_ from the massive center to the second joint, and angle *δ*_*e*_ between the massive centric line and the second link. For the sake of the comparison to the experimental results, these real parameters are presented in Table 1. As a result, *λ* is calculated below *λ* = [6.7333;3.4000;3.0000;0].

**Table 1:** Real physical parameters of a two-link robot manipulator with unknown payload.

*m*_1_	*l*_1_	*l*_*c*1_	*I*_1_	*m*_*e*_	*l*_*ce*_	*I*_*e*_	*δ*_*e*_	*e*_1_	*e*_2_
1kg	1m	1/2m	1/12kg	3kg	1m	2/5kg	0	-7/12	9.81

During the parameter identification, the reference torque trajectories *τ*_1_ = 0.1*sin*(2*ρπt*), *τ*_2_ = 0.1*cos*(2*ρπt*) (*ρ* = 0.25,0.5,1.0,2.0,4.0,5.0,5.5,6.0) are used to collect the corresponding measured data from the identified system model. The former five reference torque trajectories are prepared for the parameter estimation and the later three ones for the estimation validation.

**Algorithm 1** ELPIDSO

1: /*initialize the swarm*/

2: for *i* = 1 → *PN* **do**

3:  create particle *p*_*i*_ with dimension *D*, velocity *v*_*i*_ and position *x*_*i*_ from 1 to *PN*

4:  set *x*_*lbest*_(*i*) = *x*_*i*_

5:  calculate *fitness*(*x*_*i*_)

6: **end for**

7: set *x*_*gbest*_ = best(*x*_*lbest*_(*i*))

8: calculate inertia coefficient *w*_*pso*_, cognitive coefficient *c*_1_ and social coefficient *c*_2_

9: set maximum generation number *MaxT* and learning probability *P*_*c*_

10: /*update velocity and position with an evolutionary PID style strategy*/

11: **for** *t* = 1 → *MaxT* **do**

12:  calculate PID controller parameters: *k*_*p*_,*k*_*i*_ *and* *k*_*d*_

13:  **for** *i* = 1 → *PN* **do**

14:   */improve local best position at a given generation*/

15:   **if** *repeat*_*num*(*i*) > = 5 **then**

16:    set *repeat*_*num*(*i*) = 0, *f*_*pbest*(*i*,:) = *i*. * *ones*(1,*D*)

17:    set *fi*1 = *ceil*(*PN* * *rand*(1,*D*)), *fi*2 = *ceil*(*PN* * *rand*(1,*D*))

18:    set *fi* = (*fitness*(*fi*1) < *fitness*(*fi*2)).*fi1+(*fitness*(*fi*1) > = *fitness*(*fi*2)).*fi2

19:    set *bi* = *ceil*(*rand*(1,*D*)−1+*Pc*(*i*))

20:    **if** bi == zeros(1,D) **then**

21:     set *rc* = *randperm*(*D*), *bi*(*rc*(1)) = 1

22:    **end if**

23:    set *f*_*pbest*(*i*,:) = *bi*.*fi+(1−*bi*).*f_*pbest*(*i*,:)

24:   **end if**

25:   **for** *dimcnt* = 1 → *D* **do**

26:    set *x*_*lbest*_(*i*,*dimcnt*) = *x*_*lbest*_(*f*_*pbest*(*i*,*dimcnt*),*dimcnt*)

27:   **end for**

28:   calculate velocity *v*_*i*_ and position *x*_*i*_, according to Eqs (21) and (5)

29:   if *fitness*(*x*_*i*_) < *fitness*(*x*_*lbest*_(*i*)) **then**

30:    set *x*_*lbest*_(*i*) = *x*_*i*_

31:   **else**

32:    set *repeat*_*num*(*i*) = *repeat*_*num*(*i*)+1

33:   **end if**

34:   **if** *fitness*(*x*_*lbest*_(*i*)) < *fitness*(*x*_*gbest*_) **then**

35:    set *x*_*gbest*_ = *x*_*lbest*_(*i*)

36:    /*mutation of *x*_gbest_*/

37:    randomly select *k* between 1 and *PN*

38:    set *x*_*gbest*′_ = *x*_*gbest*_

39:    **if** *k*! = *i* **then**

40:     randomly select j between 1 and *D*, and crossover between $x_{gbest^{\prime }}^{j}$ and $x_{lbest}^{j}(k)$

41:    **else**

42:     calculate standard deviation *σ*, randomly select j between 1 and *D*, and crossover between $x_{gbest^{\prime }}^{j}$ and Eq (27)

43:    **end if**

44:    **if** *fitness*(*x*_*gbest*_) < *fitness*(*x*_*gbest*′_) **then**

45:     set $x_{gbest^{\prime }}^{j}=x_{gbest}^{j}$

46:    **end if**

47:    set *x*_*gbest*_ = *x*_*gbest*′_

48:   **end if**

49:  **end for**

50:  /*operation termination*/

51:  if goal threshold or maximum generation number *MaxT* reaches **then**

52:   break

53:  **end if**

54: **end for**

55: output results

In order to evaluate the performance of ELPIDSO, we compare four state-of-the-art evolutionary optimization algorithms including our proposed ELPIDSO, LS, GA and SPSO by identifying the parameters of the two-link robot manipulator with unknown payload. The identified result *λ* of LS is acquired by Eq (29)

$$\begin{array}{c} \lambda ={\left(Y^{T}Y\right)}^{-1}Y^{T}\tau . \end{array}$$ (29)

The four dimensional (4-D) parameters *α*,*β*,*ɛ* *and* *η* in the barycentric parameter vector *λ* are retrieved around the ranges [0, 10], [0, 5], [0, 5] and [0, 5], respectively. For GA, SPSO and ELPIDSO, population size *PN* is set at 20, maximum generation number *MaxT* is set at 15000. As is known, with the increase of population size, the median convergence characteristics of diverse evolutionary optimization algorithms for 4-D identification problem becomes faster. However, there is no obvious change on the final mean parametric results of diverse evolutionary optimization algorithms for 4-D identification problem. Their settings of other important parameters are summarized in Table 2. The above-mentioned identification criterion function is regarded as the fitness function.

**Table 2:** Parameters settings for involved evolutionary optimization algorithms.

Name	Inertia Weight	Acceleration Coefficients and Others
GA		*SP* = 30 *CP* = 0.80 *MP* = 0.10
SPSO	*w*_*pso*_(*t*) = 0.729	*c*_1_(*t*) = *c*_2_(*t*) = 1.49445
ELPIDSO	$w_{pso}(t)=\frac{1}{1+1.5\cdot e^{\left(-2.6\cdot f\right)}}f(t)=1-\frac{t}{MaxT}$	$c_{1}(t)=2.0-\frac{2.0\cdot t}{MaxT}c_{2}(t)=\frac{2.0\cdot t}{MaxT}k_{p}=e^{(w_{pso}-1)\cdot \frac{t}{MaxT}}k_{i}=\frac{e^{(w_{pso}-1)\cdot \frac{t}{MaxT}}}{1+e^{(w_{pso}-1)\cdot \frac{t}{MaxT}}}k_{d}={\left[e^{(w_{pso}-1)\cdot \frac{t}{MaxT}}\right]}^{2}$

### Parameter estimation and experimental results

We wish to test LS, GA, SPSO and ELPIDSO on the above specific fitness function with 4-D parameters *α*,*β*,*ɛ* *and* *η* for identifying the parameters of the two-link robot manipulator. To ensure the validation and accuracy of the experimental measurements, all evolutionary optimization algorithms are run 10 times on the fitness function and their final results are counted in the mean best fitness. The mean values, standard deviation of the results, and the best values are presented in tables below. When the 4-D identification problem is solved, the population size is set at 20 and the maximum FEs is set at 300,000. And in order to determine whether the results obtained by ELPIDSO are statistically different from the results generated by other evolutionary optimization algorithms, the nonparametric Wilcoxon rank sum tests are conducted between the ELPIDSO’s result and the result achieved by other evolutionary optimization algorithms for the fitness function. The h_t-tests presented in the last column of tables below are the results of t-tests. An h_t-tests of 1 indicates that the performances of the two comparative optimization algorithms are statistically different with 95% certainty, whereas an h_t-tests of 0 implies that the performances are not statistically different.

**Fig 4:**
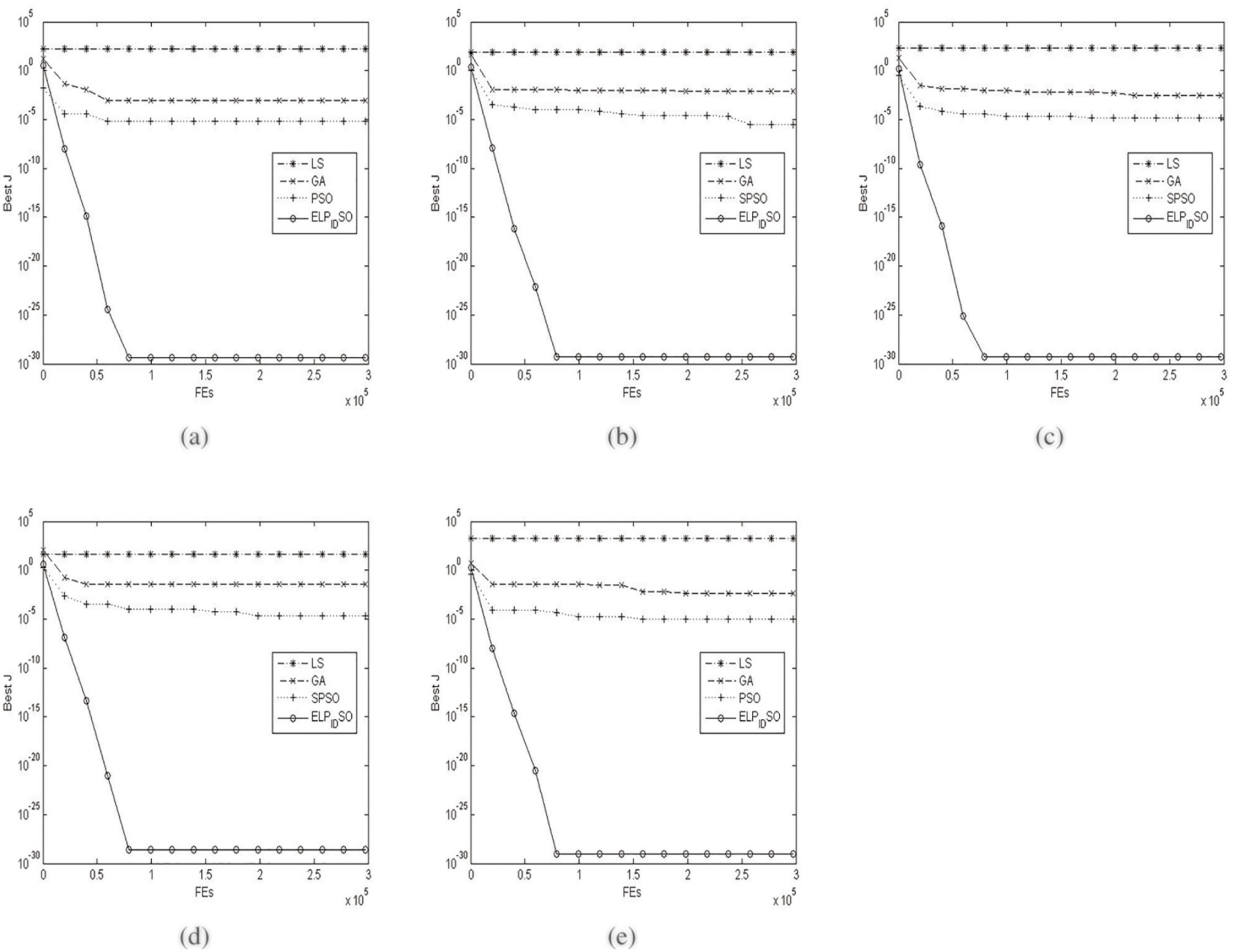
The median convergence characteristics of diverse evolutionary optimization algorithms for 4-D identification problem above. (a) *ρ* = 0.25. (b) *ρ* = 0.5. (c) *ρ* = 1.0. (d) *ρ* = 2.0. (e) *ρ* = 4.0.

Table 3 presents the means and variances of the 10 runs of the four evolutionary optimization algorithms on the above specific fitness function with its dimension 4 when *ρ* = 0.25. Table 4 presents the means and variances of the 10 runs of the four evolutionary optimization algorithms on the above specific fitness function with its dimension 4 when *ρ* = 0.5. Table 5 presents the means and variances of the 10 runs of the four evolutionary optimization algorithms on the above specific fitness function with its dimension 4 when *ρ* = 1.0. Table 6 presents the means and variances of the 10 runs of the four evolutionary optimization algorithms on the above specific fitness function with its dimension 4 when *ρ* = 2.0. Table 7 presents the means and variances of the 10 runs of the four evolutionary optimization algorithms on the above specific fitness function with its dimension 4 when *ρ* = 4.0. Table 8 presents the final means and variances of the 10 runs of the four evolutionary optimization algorithms on the above specific fitness function with its dimension 4 when *ρ* = 0.25−4.0. The best results among the evolutionary optimization algorithms are shown in bold in Tables 3, 4, 5, 6, 7 and 8. Fig 4 presents the convergence characteristics in terms of the best fitness value of the median run of diverse evolutionary optimization algorithms for the above specific fitness function with its dimension 4. The results of the proposed ELPIDSO are depicted by solid plus circle lines in Fig 4.

**Table 3:** Results of diverse evolutionary optimization algorithms for 4-D identification problem when *ρ* = 0.25.

Results		LS	GA	SPSO	ELP_ID_SO	h_t-tests
*fitness*	Mean	175.9089	0.0043	7.3845e-006	**4.5907e-030**	1
	Std. Dev	0	0.0041	4.6353e-006	**0**	
	Best	175.9089	1.9031e-004	2.3581e-006	**4.5907e-030**	
*α*	Mean	6.7471	6.3621	6.7272	**6.7333**	
	Std. Dev	0	0.5280	0.0260	**0**	
	Best	6.7471	6.3829	6.7264	**6.7333**	
*β*	Mean	3.3442	3.2138	3.3971	**3.4000**	
	Std. Dev	0	0.2667	0.0128	**0**	
	Best	3.3442	3.2231	3.3974	**3.4000**	
*ɛ*	Mean	2.9911	2.8370	2.9967	**3.0000**	
	Std. Dev	0	0.2354	0.0101	**0**	
	Best	2.9911	2.8439	3.0011	**3.0000**	
*η*	Mean	0.1841	0.0003	0	**0**	
	Std. Dev	0	0.0003	0	**0**	
	Best	0.1841	0.0000	0	**0**	

**Table 4:** Results of diverse evolutionary optimization algorithms for 4-D identification problem when *ρ* = 0.5.

Results		LS	GA	SPSO	ELP_ID_SO	h_t-tests
*fitness*	Mean	78.1332	0.0078	8.7261e-006	**5.6011e-030**	1
	Std. Dev	0	0.0057	5.8100e-006	**0**	
	Best	78.1332	6.8916e-004	2.3872e-006	**5.6011e-030**	
*α*	Mean	6.7315	5.8518	6.7101	**6.7333**	
	Std. Dev	0	0.7584	0.0227	**0**	
	Best	6.7315	5.8790	6.7221	**6.7333**	
*β*	Mean	3.4061	2.9551	3.3880	**3.4000**	
	Std. Dev	0	0.3824	0.0111	**0**	
	Best	3.4061	3.5916	3.4046	**3.4000**	
*ɛ*	Mean	3.0015	2.6089	2.9891	**3.0000**	
	Std. Dev	0	0.3360	0.0101	**0**	
	Best	3.0015	3.1679	3.0045	**3.0000**	
*η*	Mean	0.1227	0.0001	0	**0**	
	Std. Dev	0	0.0001	0	**0**	
	Best	0.1227	0.0000	0	**0**	

**Table 5:** Results of diverse evolutionary optimization algorithms for 4-D identification problem when *ρ* = 1.0.

Results		LS	GA	SPSO	ELP_ID_SO	h_t-tests
*fitness*	Mean	193.9632	0.0023	1.5972e-005	**5.4782e-030**	1
	Std. Dev	0	0.0015	7.7840e-006	**0**	
	Best	193.9632	4.1902e-004	6.1376e-006	**5.4782e-030**	
*α*	Mean	6.7336	6.0217	6.7453	**6.7333**	
	Std. Dev	0	0.4787	0.0605	**0**	
	Best	6.7336	6.7095	6.7676	**6.7333**	
*β*	Mean	3.3580	3.0337	3.4058	**3.4000**	
	Std. Dev	0	0.2469	0.0308	**0**	
	Best	3.3580	3.3884	3.4174	**3.4000**	
*ɛ*	Mean	2.9991	2.6480	3.0050	**3.0000**	
	Std. Dev	0	0.2363	0.0281	**0**	
	Best	2.9991	2.9875	3.0146	**3.0000**	
*η*	Mean	-0.1936	0.0001	0	**0**	
	Std. Dev	0	0.0001	0	**0**	
	Best	-0.1936	0.0000	0	**0**	

**Table 6:** Results of diverse evolutionary optimization algorithms for 4-D identification problem when *ρ* = 2.0.

Results		LS	GA	SPSO	ELP_ID_SO	h_t-tests
*fitness*	Mean	44.2745	0.0327	5.8134e-005	**2.7973e-029**	1
	Std. Dev	0	0.0255	2.8113e-005	**0**	
	Best	44.2745	0.0029	2.3677e-005	**2.7973e-029**	
*α*	Mean	6.7401	4.0765	6.6486	**6.7333**	
	Std. Dev	0	1.4481	0.0268	**0**	
	Best	6.7401	6.1652	6.6897	**6.7333**	
*β*	Mean	3.4217	2.0402	3.3570	**3.4000**	
	Std. Dev	0	0.7415	0.0141	**0**	
	Best	3.4217	3.1097	3.3784	**3.4000**	
*ɛ*	Mean	3.0018	1.7695	2.9602	**3.0000**	
	Std. Dev	0	0.6707	0.0134	**0**	
	Best	3.0018	2.7364	2.9808	**3.0000**	
*η*	Mean	0.0926	0.0003	0	**0**	
	Std. Dev	0	0.0002	0	**0**	
	Best	0.0926	0.0000	0	**0**	

**Table 7:** Results of diverse evolutionary optimization algorithms for 4-D identification problem when *ρ* = 4.0.

Results		LS	GA	SPSO	ELP_ID_SO	h_t-tests
*fitness*	Mean	1.9673e+003	0.0024	2.1808e-005	**8.9813e-030**	1
	Std. Dev	0	0.0021	1.7475e-005	**0**	
	Best	1.9673e+003	3.4842e-004	2.3836e-006	**8.9813e-030**	
*α*	Mean	6.7357	6.4819	6.7135	**6.7333**	
	Std. Dev	0	0.9008	0.0657	**0**	
	Best	6.7357	6.4690	6.7145	**6.7333**	
*β*	Mean	3.4008	3.2708	3.3895	**3.4000**	
	Std. Dev	0	0.4637	0.0336	**0**	
	Best	3.4008	3.2643	3.4119	**3.4000**	
*ɛ*	Mean	2.9966	2.8766	2.9892	**3.0000**	
	Std. Dev	0	0.4397	0.0318	**0**	
	Best	2.9966	2.8705	3.0086	**3.0000**	
*η*	Mean	0.6159	0.0001	0	**0**	
	Std. Dev	0	0.0001	0	**0**	
	Best	0.6159	0.0000	0	**0**	

**Table 8:** Final mean results of diverse evolutionary optimization algorithms for 4-D identification problem when *ρ* = 0.25−4.0.

Results		LS	GA	SPSO	ELP_ID_SO
*α*	Mean	6.7376	5.7588	6.7089	**6.7333**
	Std. Dev	0.0062	0.9740	0.0365	**0**
	Best	6.7336	6.4819	6.7272	**6.7333**
*β*	Mean	3.3862	2.9027	3.3875	**3.4000**
	Std. Dev	0.0333	0.4990	0.0184	**0**
	Best	3.4008	3.2708	3.3971	**3.4000**
*ɛ*	Mean	2.9980	2.5480	2.9880	**3.0000**
	Std. Dev	0.0044	0.4504	0.0169	**0**
	Best	3.0015	2.8766	2.9967	**3.0000**
*η*	Mean	0.1153	0.0002	0	**0**
	Std. Dev	0.3193	0.0001	0	**0**
	Best	0.0926	0.0001	0	**0**

From the results in Tables 3, 4, 5, 6, 7 and 8, we clearly notice that for a certain reference trajectory with a fixed *ρ*, although the identification precisions of GA, SPSO are greatly superior to those of LS in the course of identifying the parameter *η*, they exhibit worse results than LS with respect to identifying other three parameters *α*,*β* *and* *ɛ*. In addition, SPSO yields the comparatively better results than GA on estimating the four parameters. More importantly, ELPIDSO performs best for all the parameter estimation. With the increase of *ρ* from 0.25 to 4.0, SPSO still achieves better estimated results than GA whilst ELPIDSO is the best one of all the four evolutionary algorithms for the parameter estimation. From the graphs in Fig 4, one may observe that the mean fitness of LS is the worst one of all is evident. The fact reveals LS’ inferiority to other three evolutionary algorithms for the whole parameter identification. On the other hand, it is worth noting that compared to SPSO, ELPIDSO has improved a lot.

### Model validation and discussion

**Fig 5:**
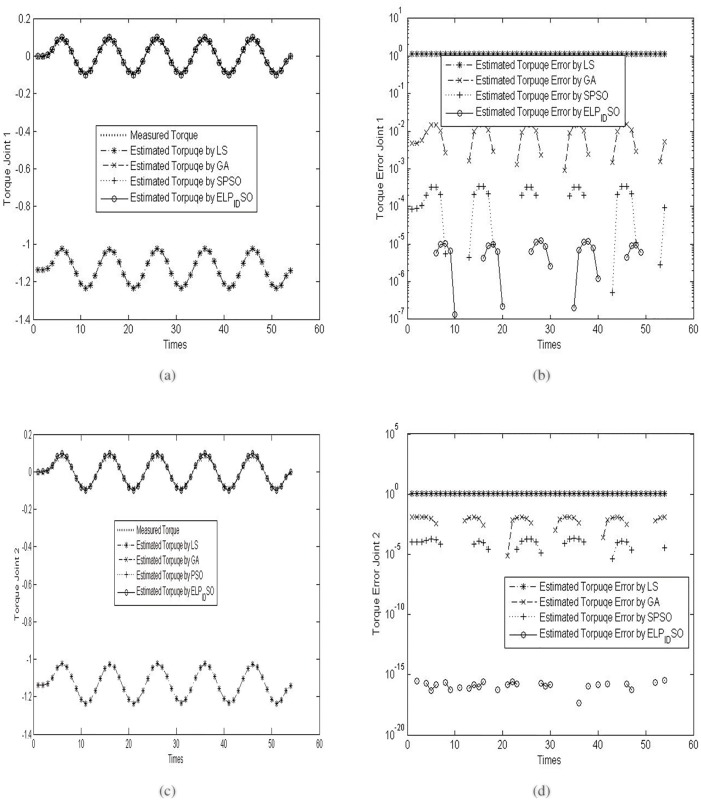
The measured torque and estimated torques by diverse evolutionary optimization algorithms on the two joints and their torque errors when *ρ* = 5.0. (a) Measured torque and estimated torques on the joint 1. (b) Estimated torque errors on the joint 1. (c) Measured torque and estimated torques on the joint 2. (d) Estimated torque errors on the joint 2.

**Fig 6:**
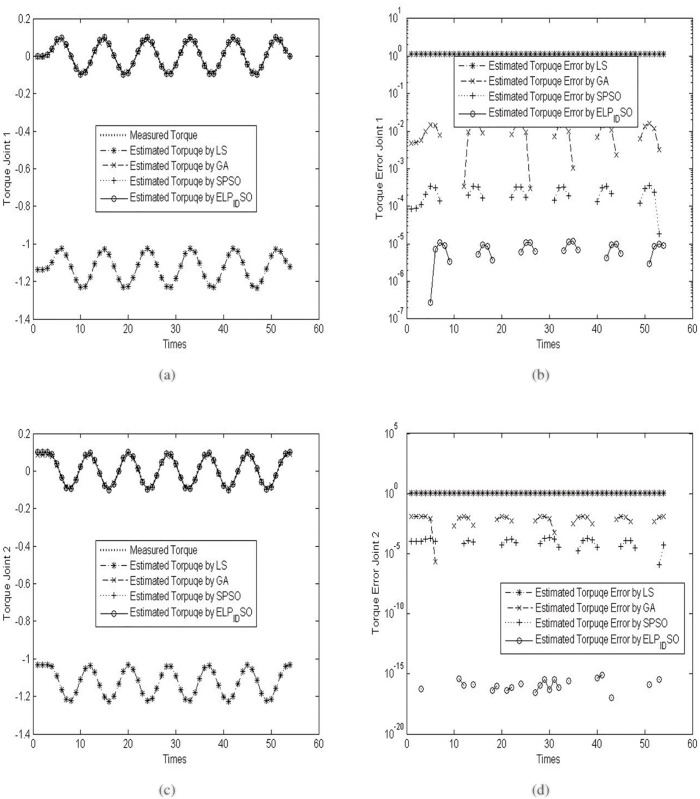
The measured torque and estimated torques by diverse evolutionary optimization algorithms on the two joints and their torque errors when *ρ* = 5.5. (a) Measured torque and estimated torques on the joint 1. (b) Estimated torque errors on the joint 1. (c) Measured torque and estimated torques on the joint 2. (d) Estimated torque errors on the joint 2.

**Fig 7:**
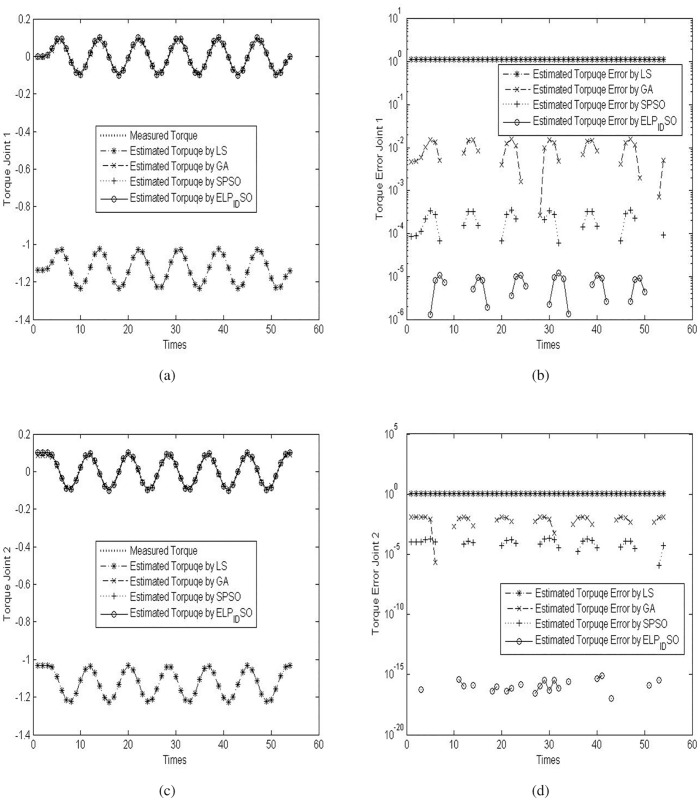
The measured torque and estimated torques by diverse evolutionary optimization algorithms on the two joints and their torque errors when *ρ* = 6.0. (a) Measured torque and estimated torques on the joint 1. (b) Estimated torque errors on the joint 1. (c) Measured torque and estimated torques on the joint 2. (d) Estimated torque errors on the joint 2.

To verify the estimation results of the four evolutionary algorithms, the three different reference trajectories with *ρ* = 5.0,5.5 *and* 6.0 are applied to the procedure of the parameter identification of the two-link robot manipulator. The concrete results of the verification experiments are presented in Figs 5, 6 and 7. Fig 5 presents the measured torque and estimated torques by diverse evolutionary optimization algorithms on the two joints and their torque errors when *ρ* = 5.0. Fig 6 presents the measured torque and estimated torques by diverse evolutionary optimization algorithms on the two joints and their torque errors when *ρ* = 5.5. Fig 7 presents the measured torque and estimated torques by diverse evolutionary optimization algorithms on the two joints and their torque errors when *ρ* = 6.0. Note that the logarithmic coordinates in Y axis are used in Figs 5(b), 5(d), 6(b), 6(d), 7(b) and 7(d) so as to highlight the discrepancies of the estimated torque errors by diverse evolutionary optimization algorithms on the two joints.

In general, two kinds of accumulated errors are used to determine which evolutionary algorithms produce more accurate estimation results. One is called absolute accumulated error (*E*_*a*_), and another is called relative accumulated error (*E*_*r*_). They are defined as follows

$$\begin{array}{c} E_{a}=\sum_{i=1}^{N}\left|\tau_{i}-\hat{\tau_{i}}\right|, \end{array}$$ (30)

$$\begin{array}{c} E_{r}=\frac{E_{a}}{\sum_{i=1}^{N}\left|\tau_{i}-\bar{\hat{\tau_{i}}}\right|}, \end{array}$$ (31)

where *N* is the number of testing samples, *τ*_*i*_ is the measured torque value of the *ith* testing sample, $\hat{\tau_{i}}$ is the estimated prediction torque value of the *ith* testing sample, and $\bar{\hat{\tau_{i}}}$ is the average of the estimated prediction torque values of the *ith* testing sample.

So we may calculate the results and obtain their corresponding relative accumulated errors of estimated torques by diverse evolutionary optimization algorithms on the two joints in Table 9. The best results among the evolutionary optimization algorithms are shown in bold in Table 9.

**Table 9:** The relative accumulated errors of estimated torques by diverse evolutionary optimization algorithms on two joints when *ρ* = 5.0,5.5,6.0.

Results		LS	GA	SPSO	ELP_ID_SO
*Joint 1*	*ρ* = 5.0	1.0018	0.1848	0.0045	**0.1601e-003**
	*ρ* = 5.5	1.0000	0.1918	0.0053	**0.1826e-003**
	*ρ* = 6.0	1.0018	1.1808	0.0045	**0.1604e-003**
	Mean	1.0012	0.1879	0.0048	**0.1677e-003**
	Std. Dev	0.0010	0.0091	0.4619e-003	**0.1290e-004**
*Joint 2*	*ρ* = 5.0	0.9989	0.1535	0.0024	**0**
	*ρ* = 5.5	1.0000	0.1686	0.0027	**0**
	*ρ* = 6.0	0.9989	0.1561	0.0024	**0**
	Mean	0.9993	0.1594	0.0025	**0**
	Std. Dev	0.0006	0.0081	0.1732e-003	**0**

As seen in Figs 5(a), 6(a) and 7(a), although the trajectories of estimated torques on the joint 1 by LS, GA, SPSO and ELPIDSO all approximate the measured torque trajectory, there exist different absolute errors among these estimated trajectories. From the graphs in Figs 5(b), 6(b) and 7(b), one may find that the absolute errors of the trajectories of estimated torques on the joint 1 by ELPIDSO is smallest while the ones of the trajectories of estimated torques on the joint 1 by LS are biggest. In addition, SPSO produces more accurate estimation results than GA. Likewise, from the graphs in Figs 5(b), 5(d), 6(b), 6(d), 7(b) and 7(d), there are similar results on the trajectories of estimated torques on the joint 2. The relative accumulated errors of estimated torques are given in Table 9. As given in Table 9, it is obvious that ELPIDSO yields the best estimation results while SPSO performs comparatively worse. The estimation results by GA are worse than those by SPSO, but better than those by LS. Despite of these, all the evolutionary algorithms can be utilized to estimate the parameters of robot manipulators.

## Conclusions and Future Work

In order to identify the parameters of robot manipulators, we present a novel variant with a time varying PID controller of particle swarm optimizers called ELPIDSO, where we attempt to use a PID controller and an elitist learning strategy to improve the performance of SPSO. Successively, ELPIDSO, together with LS, GA and SPSO, is used in the parameter identification of robot manipulators. The experimental results illustrate that due to the fact that the PID controller improves the particles’ local and global best positions information, our proposed ELPIDSO has good convergence efficiency and enhances the diversity of the swarm in comparison to SPSO. Furthermore, ELPIDSO outperforms LS, GA and SPSO in the parameter identification of robot manipulators and is regarded as a more effective tool for the future system identification in real practice.

Future work will further the elitist learning ability of ELPIDSO and the performances of PID controllers. Moreover, we will apply the proposed ELPIDSO to practical engineering applications.
